# Expression of *Ixodes scapularis* Antifreeze Glycoprotein Enhances Cold Tolerance in *Drosophila melanogaster*

**DOI:** 10.1371/journal.pone.0033447

**Published:** 2012-03-13

**Authors:** Girish Neelakanta, Andrew M. Hudson, Hameeda Sultana, Lynn Cooley, Erol Fikrig

**Affiliations:** 1 Section of Infectious Diseases, Department of Internal Medicine, Yale University School of Medicine, New Haven, Connecticut, United States of America; 2 Department of Genetics, Yale University School of Medicine, New Haven, Connecticut, United States of America; 3 Department of Cell Biology, Yale University School of Medicine, New Haven, Connecticut, United States of America; 4 Department of Molecular, Cellular, and Developmental Biology, Yale University School of Medicine, New Haven, Connecticut, United States of America; 5 Howard Hughes Medical Institute, Chevy Chase, Maryland, United States of America; University of Massachusetts Medical School, United States of America

## Abstract

*Drosophila melanogaster* experience cold shock injury and die when exposed to low non-freezing temperatures. In this study, we generated transgenic *D. melanogaster* that express putative *Ixodes scapularis* antifreeze glycoprotein (IAFGP) and show that the presence of IAFGP increases the ability of flies to survive in the cold. Male and female adult *iafgp*-expressing *D. melanogaster* exhibited higher survival rates compared with controls when placed at non-freezing temperatures. Increased hatching rates were evident in embryos expressing IAFGP when exposed to the cold. The TUNEL assay showed that flight muscles from *iafgp*-expressing female adult flies exhibited less apoptotic damage upon exposure to non-freezing temperatures in comparison to control flies. Collectively, these data suggest that expression of *iafgp* increases cold tolerance in flies by preventing apoptosis. This study defines a molecular basis for the role of an antifreeze protein in cryoprotection of flies.

## Introduction

Arthropods have evolved various strategies - including freeze tolerance, freeze avoidance, cryoprotective dehydration and rapid cold hardening - to withstand lower environmental temperatures [1], [2]. Each of these approaches is often associated with the production of specific chemicals such as antifreeze proteins [1], [2], [3], [4]. Antifreeze proteins lower the freezing point of body fluids in a non-colligative manner while not significantly affecting the melting point [5], [6], [7], [8], [9]. This property of antifreeze proteins is termed thermal hysteresis [7]. These proteins are classified into two main types, “antifreeze proteins (AFPs)” and “antifreeze glycoproteins (AFGPs)” [3], [4], [5], [8], [9] . AFPs are further divided into 4 subtypes based on the structural differences and AFGPs are divided into 8 subtypes based on the relative rates of electrophoretic migration [3], [5], [7], [8], [9]. Since their identification in marine teleost fishes from the Antarctic [6], [10], AFPs and AFGPs have been found in various arthropods [3], [4], [11], [12], [13], [14], [15].

The black-legged tick, *Ixodes scapularis*, vectors several pathogens of medical importance, including the agents of Lyme disease and human granulocytic anaplasmosis [16]. *I. scapularis* is a freeze avoiding arthropod that produces an AFGP-like agent [17]. The putative *I. scapularis* antifreeze glycoprotein (IAFGP) has a predicted molecular mass of 23.2 kDa with signal peptide sequence at N-terminus [17]. The IAFGP amino acid sequence has a high percentage of identity with several fish AFGPs [17]. The alanine-alanine-threonine repeat sequences characteristic of AFGPs and post-translational modification signals including O-glycosylation sites were evident in IAFGP. Yeast cells transfected with IAFGP showed increased viability when exposed to cold temperature [17] suggesting that expression of IAFGP confers cold tolerance when expressed in other eukaryotes.

*Drosophila melanogaster* is freeze-intolerant insect that does not possess antifreeze proteins. When exposed to temperatures approaching 0°C for extended times *D. melanogaster* experience cold-induced injury and enter into state of inactivity [18], [19], [20], [21]. The cold-induced injuries for *Drosophila* can generally be classified in to two categories [22]: injuries caused by acute stress or cold shock (short exposures to intense cold temperatures) and injuries caused by chronic or chilling stress (longer exposure to less intense cold temperatures). It is hypothesized that exposure to acute cold leads to membrane phase transitions causing immediate damage to cell membranes [23] and exposure to chronic cold leads to gradual equilibration of transmembrane ion gradients [24]. It is noted that phase transition would also result in ion equilibration [22]. However, the detailed molecular mechanisms of the cold-induced injuries and the mechanistic link between these two types of cold stress have not been established. The embryos of *D. melanogaster* also show high lethality when exposed to both acute and chronic cold stress [25], [26]. Studies have reported that both acute and chronic cold stress leads to apoptosis in some mammalian cells [27], [28], [29], [30] and *D. melanogaster* [21].

Cryopreservation of *D. melanogaster* stocks has been reported [25], [31] but is not routinely used because of the extremely high lethality during freezing and the difficulty of the procedure. Although *Drosophila* flies has a supercooling (temperature at which body fluid nucleation occurs) below −20°C, it cannot survive brief exposure at −5°C suggesting that death of flies is not caused by ice formation but rather due to cold-induced injury on membranes [25], [31]. It is unlikely that cryopreservation will be used regularly until the problem of cold-induced injury during thawing cannot be prevented. AFGPs and AFPs have been shown to confer protection against both acute and chronic cold stress to membrane components in many studies [32], [33], [34], [35], [36]. This protection has been attributed to the interaction of AFGPs and AFPs with the integral membrane components across membranes [32], [33], [34], [35], [36]. Numerous studies have therefore used different antifreeze proteins to help maintain *D. melanogaster* stocks at low temperatures. Expression of fish type III AFP in *D. melanogaster* under the control of promoters from Yolk protein genes revealed no difference in cold tolerance in the transgenic flies [37]. Transgenic *D. melanogaster* expressing the spruce budworm, *Choristoneura fumiferana* AFP gene under the control of the Actin 5C promoter had no improvement in cold tolerance [38]. However, recent studies showed that expression of beetle *Dendroides canadensis* AFP's (DAFP) conferred some degree of cold tolerance in *D. melanogaster* [39], [40]. Studies have not determined whether expression of arthropod AFGP confers cold tolerance in *D. melanogaster*. In this study we generated transgenic *D. melanogaster* that express *I. scapularis* AFGP to examine whether expression of *iafgp* confers cold tolerance in flies by preventing the problem of cold-induced injury during cryopreservation.

## Results

### Generation of *iafgp* transgenic *D. melanogaster*

**Figure 1:**
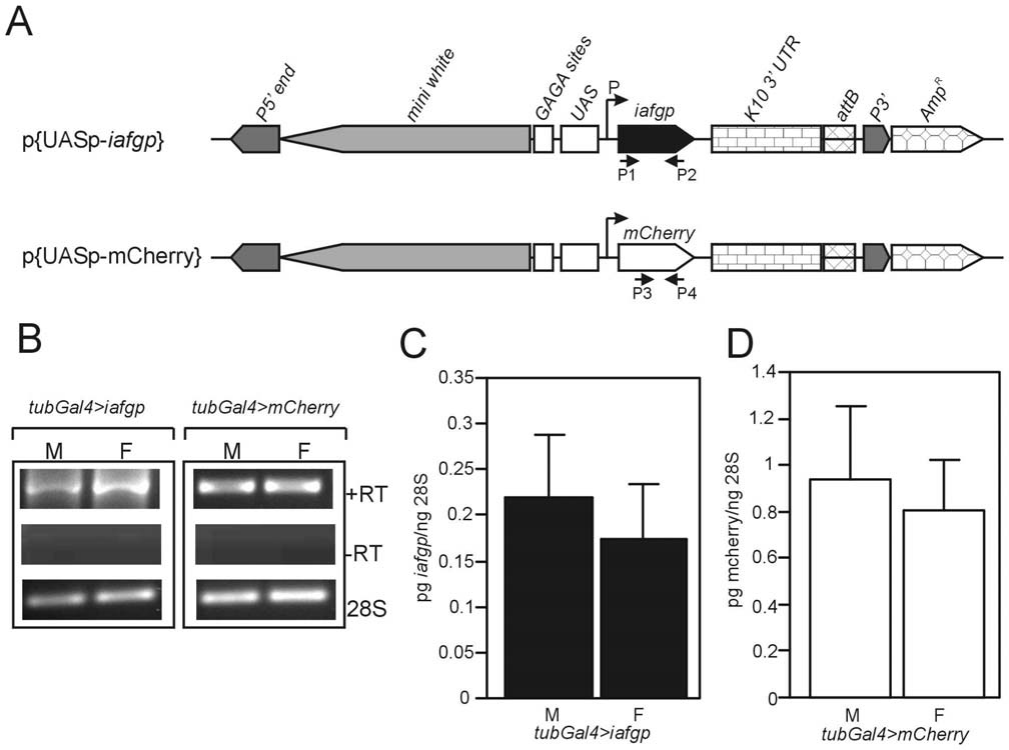
Generation of *iafgp*-transgenic *D. melanogaster*. (A) Schematic representation of constructs used to generate *iafgp*-or *mcherry*-transgenic *D. melanogaster* is shown. (B) RT-PCR results showing expression of *iafgp* or *mcherry* transcripts in *iafgp*-or *mcherry*-expressing male (M) and female (F) flies. Reactions performed with no reverse transcriptase enzyme are shown as −RT. Levels of 28S gene transcripts were used as controls. Quantitative PCR results showing levels of *iafgp* (C) or *mcherry* (D) transcripts normalized to the levels of 28S gene transcripts in *iafgp*-or *mcherry*-expressing male and female flies.

IAFGP plays a critical role in the ability of *I. scapularis* to survive in the cold [17]. We tested whether the expression of *iafgp* could alter the cold-tolerance of *D. melanogaster*, an arthropod commonly used as a model for numerous scientific studies. A transformation construct (*p{UASp-iafgp}*) was made with *iafgp cDNA* using a promoter under control of the Gal4 upstream activating sequence (Fig. 1A). As a control, a construct carrying the *mCherry* gene was generated (Fig. 1A). These constructs were transformed into *D. melanogaster* embryos and transgenic fly lines were generated as described [41], [42].

Flies were then crossed with *tubulin-Gal4*-expressing flies and adult progeny were analyzed for expression of the *iafgp* or *mcherry* genes. RT-PCR analysis confirmed expression of the *iafgp* or *mcherry* genes (Fig. 1B). Quantitative PCR (Q-PCR) analysis showed no differences in the expression of *iafgp* or *mcherry* in male and female *D. melanogaster* (Fig. 1C & D).

### Expression of *iafgp* increases cold tolerance of *D. melanogaster*

**Figure 2:**
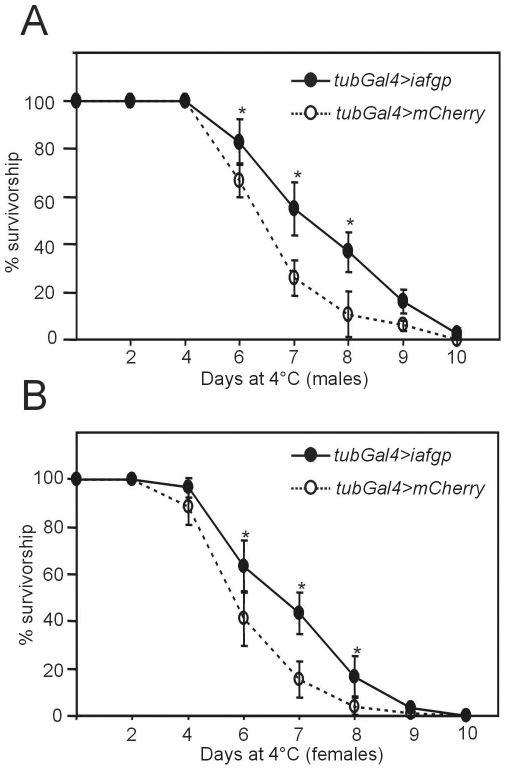
Expression of *iafgp* increases cold tolerance in *D. melanogaster* adult flies. Survival of male (A) and female (B) *iafgp-or mcherry*-expressing flies after cold treatment at 4°C for 2, 4, 6, 7, 8, 9 and 10 days. Mean value from six independent experiments with 25 flies/group/time point is shown. Error bars indicate (±) standard deviation from the mean value. Statistical significance (P<0.05) is indicated with an asterisk and was calculated using ANOVA and Tukey's post test.

To determine whether expression of *iafgp* increased the cold tolerance of *D. melanogaster*, we first performed studies with adult flies. Male and female flies were separately analyzed because of reported differences in their cold tolerance [43]. We incubated *iafgp* or *mcherry*-expressing flies at 4°C and analyzed survival at various time points (Fig. 2A & B). The lethal time-point _50_ (LT_50_) at 4°C for control flies (*tubGal4>mCherry* flies) was 6–7 days for males and 4–6 days for females (Fig. 2A & B). The *tubGal4>iafgp* flies had an increased LT_50_ (7–8 days for males and 6–7 days for females) compared with control flies (P<0.05, Fig. 2A & B). Significantly (∼two–four fold, P<0.05) increased survival rates for *iafgp*-expressing flies were evident at days 6, 7 and 8 time points for both males and females in comparison to controls (Fig. 2A & B). None of the flies survived when incubated more than 10 days at 4°C (Fig. 2A & B).

### Expression of *iafgp* increases cold tolerance of *D. melanogaster* embryos

**Figure 3:**
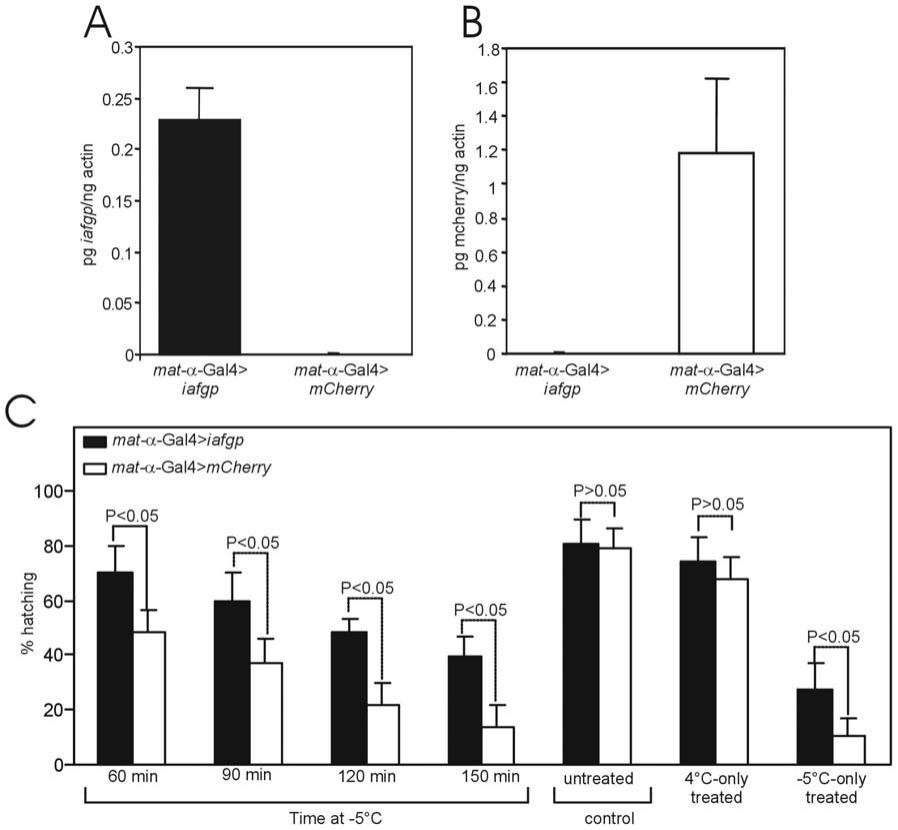
Expression of *iafgp* increases cold tolerance in *D. melanogaster* embryos. Quantitative RT-PCR results showing levels of *iafgp* (A) or *mcherry* (B) transcripts normalized to actin transcript levels in embryos collected from *iafgp-or mcherry*-expressing flies. Shown are the results from three independent experiments. (C) Embryos were collected from *iafgp-or mcherry*-expressing flies after cold treatment at −5°C for 60, 90, 120 and 150 min. Percentage hatching of embryos to larvae was calculated as described in methods. Mean value from eight independent experiments with 20 embryos/group/time point is shown. Error bars indicate (+) standard deviation from the mean value. As controls, embryos incubated at 25°C (untreated) or 4°C for 4 h or cold treated at −5°C for 120 min without 4°C pause were included. Statistical significance (P<0.05) was calculated using ANOVA and Tukey's posttest.

As IAFGP increased the ability of adult flies to survive in the cold, we determined whether expression of *iafgp* increases the cold tolerance of *D. melanogaster* embryos. Q-PCR analysis confirmed the presence of *iafgp* or *mcherry* transcripts in *D. melanogaster* embryos (Fig. 3A & B). Cold tolerance assays with 12–16 h embryos were performed as described in the methods. We incubated embryos generated from *mat-α-Gal4>iafgp* or *mat-α-Gal4>mCherry* females at −5°C for different lengths of time, restored them to 4°C for 4 h and 25°C for 48 h, and analyzed the hatching of embryos (Fig. 3C). Hatching assays showed that embryos with IAFGP were more resistant to cold shock than controls with an increased ability to hatch into larvae at all time points (2–3 fold, P<0.01) in comparison to controls (Fig. 3C). Embryos with IAFGP were also more resistant (2–3 fold, P<0.05) to −5°C cold shock for 120 min in comparison to control even without 4°C incubation during recovery (Fig. 3C). No differences in hatching between embryos carrying IAFGP and controls were noted in the untreated groups and embryos that were treated at 4°C for 4 h (P>0.05, Fig. 3C). These results show that expression of *iafgp* increases the cold tolerance of *D. melanogaster* embryos.

### Expression of *iafgp* protects *D. melanogaster* cells from cold stress and apoptotic cell death

**Figure 4:**
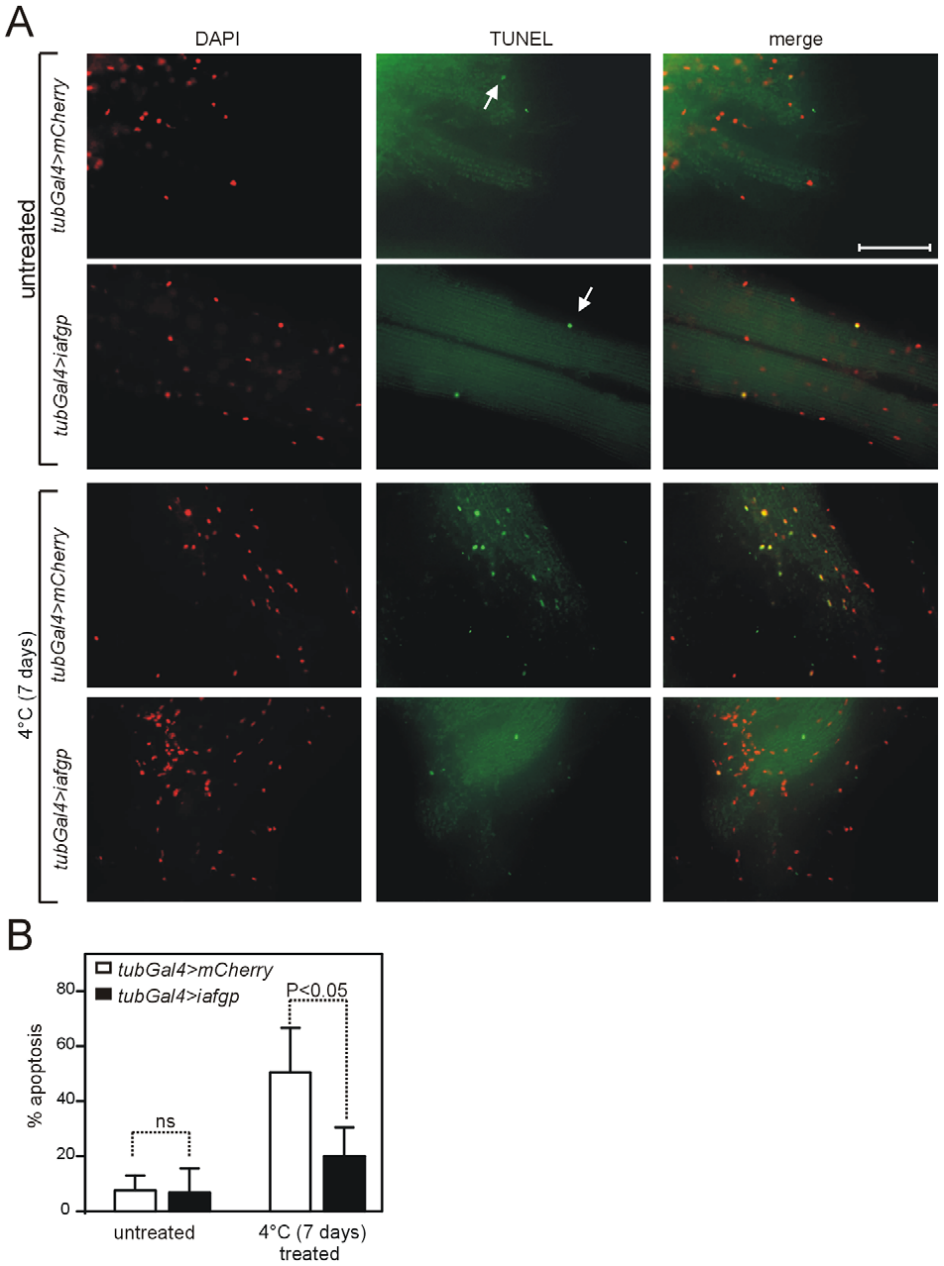
Expression of *iafgp* protects *D. melanogaster* flight muscles from cold stress. (A) Shown are the TUNEL/DAPI stained and merged microscopic representative images of flight muscles dissected from *iafgp*-or *mcherry*-expressing adult female flies after cold shock at 4°C for 7 days. High proportion of TUNEL positive nuclei were evident in *mcherry*-expressing flies in comparison to *iafgp*-expressing flies. Flight muscles isolated from untreated flies incubated at 25°C served as controls. Arrow head points to representative TUNEL positive nuclei. Scale bar represents 50 micron. (B) Quantification of percentage of apoptosis as determined by TUNEL assay in (A). Error bars represents (+) standard deviation from the mean value. ns indicates not significant. Statistical significance (P<0.05) was calculated using Student's t test.

Studies have reported that cold induces apoptosis in both mammalian cells and *Drosophila* [21], [27], [28], [29], [30], [44], [45]. To characterize the functional role of IAFGP in cold tolerance of *D. melanogaster*, we determined whether expression of *iafgp* protects *Drosophila* cells from apoptotic cell death. A TUNEL assay examined whether apoptosis occurred in response to injury caused by exposure to the cold in flight muscle, which has previously been shown to undergo increased apoptosis in response to cold treatment [21]. TUNEL staining was performed with flight muscles dissected from *iafgp* or *mcherry*-expressing female flies incubated for 7 days at 4°C as described in methods. An increased number of TUNEL positive nuclei were observed following cold treatment in flight muscles from *mCherry*-expressing flies in comparison to flight muscles from *iafgp*-expressing flies (Fig. 4A). Furthermore, quantification of images (as described in methods) revealed significantly increased number of TUNEL positive nuclei in flight muscles from *mCherry*-expressing flies (∼50%, P<0.05) incubated for 7 days at 4°C in comparison to flight muscles from *iafgp*-expressing flies (∼20%) (Fig. 4B). No difference was seen between flight muscles isolated from untreated *iafgp-* and *mCherry*-expressing flies (Fig. 4A & B). Collectively, these results show that expression of *iafgp* protects *D. melanogaster* cells from apoptotic cell death during cold treatment and thereby increases cold tolerance in flies.

**Figure 5:**
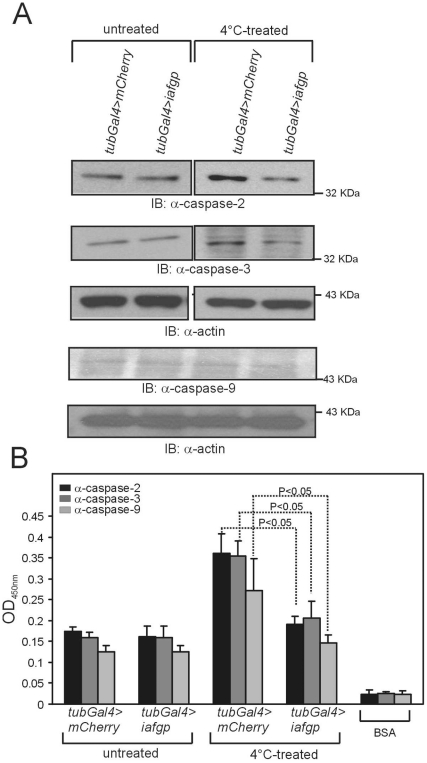
IAFGP confers resistance to cold stress in *D. melanogaster* by preventing apoptotic progress. (A) Immunoblot analysis using antibodies to caspase -2, -3 and -9 is shown. Total lysates were prepared from *iafgp*- or *mcherry*-expressing flies after cold shock at 4°C for 6 days. Untreated flies incubated at 25°C served as experimental controls. Levels of actin served as loading control. (B) ELISA analysis using antibodies to caspase-2, -3 and -9 is shown. Total lysates were prepared from *iafgp*- or *mcherry*-expressing flies after cold shock at 4°C for 6 days and coated onto ELISA plates and assayed in triplicates as described in methods. Bovine serum albumin (BSA) was used as control sample. Error bars represent (+) standard deviation from the mean value.

As IAFGP protects *D. melanogaster* flight muscle cells from cold temperature stress and apoptotic cell death (Fig. 4), we analyzed whether this effect is due to inhibition of apoptosis genes. We performed immunoblotting of total lysates from *iafgp* or *mcherry*-expressing male flies to examine the expression of both “initiator” caspases (Caspase-2 and Caspase-9) and an “executioner” caspase (Caspase-3), which are central players in the apoptotic pathway [46], [47], [48], [49]. Our immunoblotting assays using commercially available antibodies generated against human caspase-2, -3 or -9 detected strong bands at the sizes ∼35 KDa, ∼38 KDa and ∼47 KDa respectively in flies incubated at 25°C (untreated control group) or 4°C for 6 days (treated group). In the untreated group the intensity levels of all three bands were same in both *iafgp* and control transgenic flies (Fig. 5A, Table S1). However, in the 4°C-treated group, we found reduced levels of all three bands in *iafgp*-expressing flies compared to the controls (Figure 5A, Table S1). Enzyme linked immunosorbant assay (ELISA) using these antibodies yielded similar results (Figure 5B). Collectively, based on these studies we hypothesize that expression of IAFGP increases cold tolerance in flies at least in part, by preventing the initiation of apoptosis.

## Discussion

Attempts to confer cold tolerance in *D. melanogaster* using fish AFP type antifreeze proteins have been unsuccessful due to the decreased activity of fish AFPs when expressed in arthropods [37], [38]. Arthropod AFPs are believed to have 10–30 times higher activity than fish AFPs [50], [51]. The effect of arthropod AFGP type antifreeze protein on *D. melanogaster* cold tolerance remained unknown. We have now generated transgenic *D. melanogaster* that express *I. scapularis* AFGP and showed that this arthropod AFGP-like antifreeze protein confers cold tolerance in flies.

Cold stress at non-freezing temperatures results in transient increases in cellular membrane permeability, leakage and cellular damage [19], [20]. Studies have shown that stabilization of cellular membranes is an important factor for *D. melanogaster* to survive cold stress [19], [20]. Overgaard et al., found increased survivorship of *D. melanogaster* upon rapid cold hardening (RCH), a protective effect gained by gradual cooling, at temperatures slightly below freezing [52]. Their study indicated that increased survival of *D. melanogaster* after RCH is probably due to an increase in unsaturated fatty acids in the membrane phospholipids. Although the RCH response was shown to prevent lethal injury caused by acute cold exposure in many insects [52], [53], [54], studies have not explored whether RCH also imparts increased tolerance to chronic cold exposures. More recently, increases in membrane cholesterol levels were associated with increased cold tolerance in *D. melanogaster* [55]. Human platelets maintained viability when stored with fish AFGP for 21 days at 4°C [34]. In the Rubinsky et al., study cow and pig oocytes were chilled to 4°C and re-warmed in the presence of AFGP at 37°C [33]. Results from their study showed that AFGP helped maintain the viability and membrane potential of these cells [33]. Furthermore, AFP type antifreeze proteins protect cells from cold shock by inhibiting leakage across membranes containing unsaturated chloroplast galactolipid [35], [36]. Collectively, these studies imply that antifreeze proteins can protect cells from both cold shock and chilling injuries. In our experiments, transgenic *D. melanogaster* adult flies and embryos exhibited higher survival and hatching rates respectively after cold stress (Fig. 2 & 3), suggesting a role for IAFGP in preventing injuries to the membrane caused due to both acute and chronic cold exposures.

Loss of membrane stability is a feature of cells undergoing apoptosis [47], [48]. We therefore analyzed whether IAFGP prevents the progression of apoptosis in flies. Several studies have shown that apoptosis was induced upon both acute and chronic cold exposures [21], [44], [45]. In addition to the loss of membrane stability, apoptosis is characterized by condensation of the nuclear chromatin and shrinkage of the cytoplasm [46], [47], [48], [49]. Our finding that flight muscles isolated from *iafgp*-expressing females after cold stress have less TUNEL positive nuclei than control flies (Fig. 4) reflects the role of IAFGP in preventing apoptosis. Caspases such as caspase-2, -3 and -9 play important roles in apoptosis [48], [49], [56]. There are seven caspase-like molecules in *D. melanogaster* that are termed as DCP-1, DREDD/DCP-2, DRICE, DRONC, DECAY, DAMM and STRICA/DREAM [57]. Compared to mammalian caspases, less is known about the biology of *D. melanogaster* caspase-like proteins [57]. DECAY, DRICE and DCP-1 shows high homology to mammalian caspase-3 [57]. DREDD shows homology to caspase 2 and DRONC is a functional homologue of Caspase-9 [57]. We postulate that the antibodies directed against human caspases-2, -3 or -9 possibly detected the respective *D. melanogaster* homologues. However, it remains to be clarified whether presence of IAFGP has any impact on caspase activation in flies.

Three scenarios may be envisioned for the role of IAFGP in the cold tolerance. Firstly, IAFGP may directly stabilize membranes by preventing denaturation of macromolecules, blocking ion fluxes across membranes or by inhibiting the leakage of liposomes. Secondly, IAFGP may directly block apoptosis and stabilize the membranes. Thirdly, IAFGP may work in concert with other cold tolerance strategies such as RCH to maintain stability of the membrane by blocking apoptosis progress. With any of these models, our finding that *iafgp*-expressing *D. melanogaster* show better survival at cold temperature suggests the cold tolerance mechanism of flies can be augmented. Co-expression of other synergistic components such as *D. canadensis* AFP's, in addition to *I. scapularis* IAFGP may further improve cold tolerance in flies. This study not only furthers our understanding of the molecular basis of antifreeze protein-mediated cryoprotection in flies but also provide information on the mechanisms that are important in the stabilization of cellular membranes at low temperatures.

## Materials and Methods

### D. melanogaster strains

*D. melanogaster* cultures were maintained using standard procedures [58]. Ubiquitous expression of *p{UASp-iafgp}* and *p{UASp-mCherry}* transgenes was achieved by crossing to *P{tub-Gal4}LL7* [59]. Embryos with a maternal contribution of either *iafgp* or *mCherry* were collected from mothers expressing the transgenes under the control of *p{mat-α-Gal4::VP16}*, a strong maternal Gal4 driver [60].

### Construction of transgenes and generation of transgenic lines

The *iafgp* coding region was amplified from pGEMT-*iafgp* [17] using oligonucleotides P1 5′ GGGGTACCGCCGCCACCATGACGACTCTGCTTCGTCTGACT 3′ and P2 5′ CGGGATCCCTACGCAGCCGCCGTAGCTGCCGT 3′. The polymerase chain reaction (PCR) product was digested with KpnI and BamHI restriction enzymes and cloned into KpnI-BamHI digested pYS041, a UASp vector [61] modified to include a phiC-31 *attB* integration sequence downstream of the 3′-UTR (Y. Shimada and Lynn Cooley). Schematic representation of the final transgenic construct (*p{UASp-iafgp}*) is shown in Figure 1. *p{UASp-mCherry}* was generated by cloning the TAPmCherry fusion described previously [41] as a KpnI-BamHI fragment into pYS041. These two phiC31 integration constructs were purified using Qiagen miniprep kit (Qiagen, USA) and micro-injected by Duke University Model System Genomics into the AttP2 landing site located at 68A4 [62]. Total genomic DNA from transgenic flies was extracted using Qiagen DNeasy Blood and tissue kit (Qiagen, USA) and analyzed for the presence of transgenes. Oligonucleotides P1 & P2 were used to detect *iafgp* transgene and P3 5′ GCAGTGAGCAAGGGCGAGGA 3′ and P4 5′ GGGGAAGGACAGCTTCAAGTAGT 3′ were used to detect *mcherry* transgene.

### Adult fly cold tolerance assays

To generate experimental flies, *iafgp* or *mcherry* transgenic male flies were crossed with virgin *p{tub-Gal4}LL7* female flies. Male and female flies that hatched from this cross were separated and reared up to 3 days at 25°C. One to three days old post-emergence male and female flies were transferred to fresh 25 ml plastic vial containing fly solidified food medium and placed at 25°C for an additional day. These two to four day old post-emergence flies were then split into seven groups with 25 flies/group in individual 25 ml plastic vials containing fly food medium and incubated at 4°C in the cold room. In order to maintain constant temperature parameter throughout our independent experimental procedures, incubations were carried out in cold room with no control on light cycle. New plastic vials with solidified food medium were used in order to prevent flies being extensively stuck in the food medium during or after cold treatment procedure. After cold treatment at 4°C for 2, 4, 6, 7, 8, 9 and 10 days, plastic vials containing flies were shifted to 25°C for 24 hours and analyzed for fly survival rate. Those flies that are still lightly adhered to the solid food medium were gently removed with brush or by gently tapping the vial and analyzed for survival. Flies able to walk or fly or right themselves were considered viable. Percentage of fly survival at 4°C was determined for each time point and survival curves were generated from six independent experiments with 25 flies/group/timepoint. Statistical significance was calculated using ANOVA and Tukey's multiple comparison tests.

### Embryo cold tolerance assays

Virgin *p{mat-α-Gal4::VP16}* female flies were crossed with *iafgp* or *mcherry* transgenic male flies. Male and females adult flies from this cross were reared together up to two days post emergence in 250 ml plastic flask containing fly food and yeast paste. These flies were then placed together in small embryo collection cages (Genessee Sceintific, USA) with 60 mm petri dish (Becton Dickinson, USA) containing apple juice agar medium. After four hours of embryo collection, petri dishes containing embryos were removed from the embryo collection cages and stored at 25°C for additional 12 hours to allow embryonic development to 12–16 h stage. These 12–16 h embryos containing IAFGP or mCherry were then split into 5 groups with 20 embryos/timepoint/group and placed side-by-side on fresh 35×10 mm petri dish containing apple juice agar culture media. Petri dishes containing embryos are covered with lids and were immediately shifted to centrifuge pre-set to −5°C. Petri dishes containing embryos were placed in the centrifuge with the bottom of the petri dish facing metal surface. After cold treatment at −5°C for 60, 90, 120 and 150 minutes, petri dishes containing embryos were shifted to 4°C for 4 hours followed by 25°C for 48 hours to reacclimatize embryos. As controls, embryos incubated at 25°C (untreated) for 48 h or 4°C for 4 h or cold treated at −5°C for 120 min without 4°C pause were included in the study. Percentage viability was calculated based on the number of embryos hatched to the total number of embryos placed in each petri dish. Eight independent experiments were performed and statistical significance was calculated using ANOVA and Tukey's multiple comparison tests.

### Quantitative Real-time PCR (QRT-PCR) Analysis

Total RNA from adult flies or embryos was extracted using Qiagen RNeasy mini kit (Qiagen, USA) and converted to cDNA using BioRad cDNA synthesis kit (BioRad, USA). The generated cDNA was used as template for the amplification of *iafgp* or *mcherry* mRNA. QRT-PCR was performed using iQ-SYBR Green Supermix (Biorad, USA). QRT-PCR reaction conditions were followed as described [17], [63]. Standard curve was prepared using 10-fold serial dilutions of known quantities of *iafgp* or *mCherry* amplicons. As an internal control and to normalize the amount of template, *actin* or 28S amplicons were quantified and QRT-PCR reactions were performed as described [63]. The levels of *iafgp* and 28S mRNA were quantified using oligonucleotides as described [17], [64], actin mRNA levels were quantified using oligonucleotides 5′ CCCCAAGGCCAACCGTGA 3′ and 5′ CAAATCGCGACCAGCCAGA 3′.

### TUNEL assay and microscopy

Flight muscles were dissected from female *iafgp*- or *mCherry*-expressing flies after cold shock at 4°C for 7 days and from untreated group (25°C incubation). Dissection of flight muscles was performed in in PBS buffer and fixation in 4% formaldehyde for 20 min, followed by washing and incubations in PBS supplemented with 0.1% Triton X-100 (PBTx) as described [41]. Tissues were then permeabilized in 100 mM Citrate/0/1% Triton-X-100 for 30 min at 65°C and rinsed with PBS containing 0.5% Triton X-100 (PBTx5). Flight muscles were then processed for TUNEL assay using In situ cell death detection kit, Fluorescein (Roche, USA). Briefly, tissues were incubated in TUNEL Assay buffer at 25°C for 30 min followed by incubation at 37°C for 30 min. TdT enzyme was added into the reaction buffer and tissues were further incubated for 12 hours at 37°C followed by washing with PBTx. Tissues were then fluorescently labeled with 4′6-diamidino-2-phenylindole (DAPI). DAPI and TUNEL stained samples were viewed under inverted microscope (Axiovert 200; Car Zeiss Inc.,) equipped with a confocal imaging system (CARVII; BioVision) and a camera (CoolSNAP HQ2; Photometrics). Images were later edited using ImageJ (National Institutes of Health) and assembled with Illustrator CS2 (Adobe) and CorelDraw software. Percentage apoptosis was calculated based on the number of TUNEL positive nuclei to the total number of DAPI stained nuclei in each image and data was plotted using Microsoft Excel. A total of 15–30 images were analyzed in each group.

### Immunoblotting

Total lysates from male *iafgp*- or *mCherry*-expressing flies cold shocked at 4°C for 6 days or from flies placed at 25°C without cold shock were extracted and analyzed for the levels of apoptotic markers. About 10 flies were homogenized using Pellet pestle cordless motor and pellet pestle (Fisher Scientific, USA) in lysis buffer 50 mM Hepes, pH 7.5, 150 mM NaCl, 5 mM EGTA, 0.5% Triton X-100, 10% glycerol supplemented with 1×complete protease inhibitor mix (Roche, USA). Protein concentrations were measured using Bio-Rad protein assay reagent (BioRad, USA). Proteins were separated on SDS-PAGE and analyzed by Western blotting as described [65]. Antibodies directed against actin [65], Caspase-2 (rat monoclonal Anti-Caspase 2, Clone 10C6, Millipore, USA), Caspase-3 (rabbit polyclonal Anti-Caspase 3, Millipore, USA) and Caspase-9 (rabbit monoclonal Anti-Caspase 9, Abcam, USA) were used from commercial sources. Depending on the primary antibodies the membrane was incubated with either 5% BSA or milk (according to the manufacturer's instructions) in Tween-20-Tris-buffered saline to bind non-specific sites. Anti-mouse/rat/rabbit HRP conjugated IgG secondary antibodies (Sigma) were used following primary antibody incubation and enhanced chemiluminescence's detection of antibody binding was performed with the ECL™ Western blotting detection system (GE Heathcare). Western blot images were then analyzed and quantified in Adobe Photoshop following the method as described [66]. The relative intensity of each band on the immunoblots was calculated with respect to the actin levels. Quantification analysis of the blots is shown in Table S1.

### ELISA

ELISA was performed to detect apoptotic proteins in the total lysates prepared from *iafgp*-or *mcherry*-expressing flies. Five micrograms (100 µl) of total lysates from male *iafgp*- or *mCherry*-expressing flies cold shocked at 4°C for 6 days or from flies placed at 25°C without cold shock were coated onto ELISA plates (NUNC, USA) and incubated at 4°C overnight. Control wells were coated with 1% Bovine serum albumin in PBS. Following incubation, samples were blocked and processed for respective primary antibodies prepared in PBS-Tween20 (0.05%) buffer. Primary antibodies used are mentioned in immunoblotting section of the methods. Reactions were carried out as mentioned [63]. Samples were then incubated with respective secondary antibodies conjugated to horseradish peroxidase followed by treatment with TMB microwell peroxidase (KPL) for color development. The reactions were stopped after 30 min using TMB stop solution (KPL) and optical density (OD) was read at 450 nm.

### Statistical analysis

Errors bars define (+) standard deviation from the mean values. Statistical significance of differences observed in data sets was analyzed using GraphPad Prism4 software and Microsoft Excel. For data with small variation and to compare two means, the non-paired Student *t* test was performed. In adult and embryo cold tolerance assays, one-way analysis of variance (ANOVA) followed by Tukey's posttest was performed to compare multiple means. Tukey's post test was performed with confidence interval set at 95% to find means that are significantly different from one another. P values of <0.05 were considered significant in all tests. Wherever necessary, statistical test and P values used are reported.

## Supporting Information

Table S1 **Quantification of Western blots shown in** **Figure 5** **.**
(DOC)
