# Supplementary material for: Expression of Ixodes scapularis Antifreeze Glycoprotein Enhances Cold Tolerance in Drosophila melanogaster
Source: PLoS One. 2012 Mar 13;7(3):e33447. doi: 10.1371/journal.pone.0033447 (PMC3302814; doi:10.1371/journal.pone.0033447)
Supplement: Table S1 — Quantification of Western blots shown in Figure 5 . (DOC) [file pone.0033447.s001.doc]

**Supporting Information**

**Supporting Table S1**

**Table S1: Quantification of Western blots shown in Figure 5**

| **Figure** | **Panel** | **sample^a^** | **Relative intensity^b^** | **Fold increase^c^** |
| --- | --- | --- | --- | --- |
| 5 | α-caspase-2 | *tubGal4>mCherry* (untreated) | 0.54441341 | 0.906391095 |
|  |  | *tubGal4>iafgp* (untreated) | 0.600638524 |  |
|  |  | *tubGal4>mCherry* (4°C-treated) | 0.810561572 | 2.691483889 |
|  |  | *tubGal4>iafgp* (4°C-treated) | 0.301157876 |  |
|  | α-caspase-3 | *tubGal4>mCherry* (untreated) | 0.414580004 | 0.99878131 |
|  |  | *tubGal4>iafgp* (untreated) | 0.415085865 |  |
|  |  | *tubGal4>mCherry* (4°C-treated) | 1.010852397 | 2.195459027 |
|  |  | *tubGal4>iafgp* (4°C-treated) | 0.460428723 |  |
|  | α-caspase-9 | *tubGal4>mCherry* (untreated) | 0.349210412 | 1.031305406 |
|  |  | *tubGal4>iafgp* (untreated) | 0.338610086 |  |
|  |  | *tubGal4>mCherry* (4°C-treated) | 0.617677955 | 2.206319764 |
|  |  | *tubGal4>iafgp* (4°C-treated) | 0.279958492 |  |

a: names correspond to sample names shown in the Figure

b: relative intensity is calculated as mentioned in methods and was used to calculate fold differences

c: fold difference is calculated between *tubGal4>mCherry* (untreated) versus *tubGal4>iafgp* (untreated) and *tubGal4>mCherry* (4°C-treated) versus *tubGal4>iafgp* (4°C-treated).
